# The Pharmaceutical Industry in 2020. An Analysis of FDA Drug Approvals from the Perspective of Molecules

**DOI:** 10.3390/molecules26030627

**Published:** 2021-01-25

**Authors:** Beatriz G. de la Torre, Fernando Albericio

**Affiliations:** 1 KRISP, College of Health Sciences, University of KwaZulu-Natal, Durban 4001, South Africa; 2 School of Chemistry and Physics, University of KwaZulu-Natal, Durban 4001, South Africa; 3 Institute for Advanced Chemistry of Catalonia (IQAC-CSIC), 08034 Barcelona, Spain; 4 CIBER-BBN, Networking Centre on Bioengineering, Biomaterials and Nanomedicine, Department of Organic Chemistry, University of Barcelona, 08028 Barcelona, Spain

**Keywords:** antibodies, antibody–drug conjugate, API, biologics, CBER, CDER, chemical entities, COVID-19, drug discovery, fluorine-based drugs, natural products, nitrogen aromatic heterocycles, oligonucleotides, peptides, TIDES, small molecules

## Abstract

Although the pharmaceutical industry will remember 2020 as the year of COVID-19, it is important to highlight that this year has been the second-best—together with 1996—in terms of the number of drugs accepted by the US Food and Drug Administration (FDA). Each of these two years witnessed the authorization of 53 drugs—a number surpassed only in 2018 with 59 pharmaceutical agents. The 53 approvals in 2020 are divided between 40 new chemical entities and 13 biologic drugs (biologics). Of note, ten monoclonal antibodies, two antibody–drug conjugates, three peptides, and two oligonucleotides have been approved in 2020. Close inspection of the so-called small molecules reveals the significant presence of fluorine atoms and/or nitrogen aromatic heterocycles. This report analyzes the 53 new drugs of the 2020 harvest from a strictly chemical perspective, as it did for those authorized in the previous four years. On the basis of chemical structure alone, the drugs that received approval in 2020 are classified as the following: biologics (antibodies, antibody-drug conjugates, and proteins); TIDES (peptide and oligonucleotides); natural products; fluorine-containing molecules; nitrogen aromatic heterocycles; and other small molecules.

## 1. Analysis

**Figure 1:**
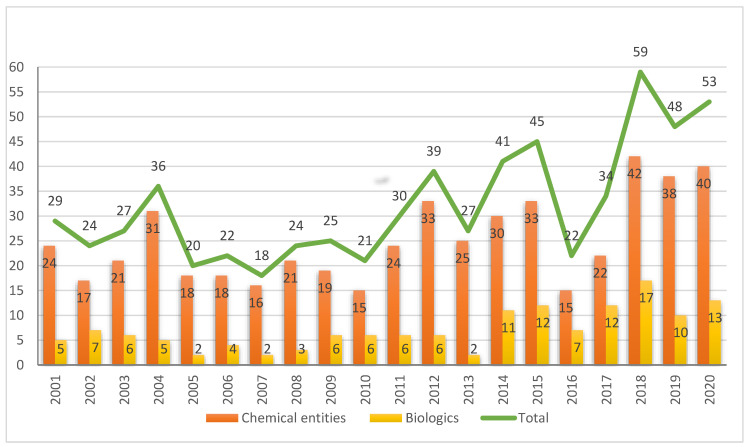
Drugs (New Chemical Entities and Biologics) approved by the FDA in the last two decades [1].

From the context of health, 2020 has been without doubt the most difficult year in living memory. In this regard, it will be remembered as the year of coronavirus disease 2019 (COVID-19), which has changed our everyday lives and also the way in which the stakeholders involved in the health ecosystem conduct their business. The emergence of COVID-19 at the beginning of 2020 brought about unprecedented events. The pharmaceutical industry has responded rapidly, gearing itself up for the development of vaccines to tackle the pandemic; the two most important health agencies worldwide, namely the Food and Drug Administration (FDA) and the European Medicines Agency (EMA), have approved the two first vaccines against COVID-19, and, towards the end of the year, the first people were vaccinated. This extraordinary sequence of events should not overshadow the outstanding year that 2020 (also referred to as “this year” herein) has been with respect to the approval of new drugs. In this regard, the FDA’s Center for Drug Evaluation and Research (CDER) has approved 53 new drugs this year [1], which is the second-best harvest—together with 1996— and one that is very close to the record-breaking 59 approvals in 2018 and slightly superior to the 48 approvals in 2019. These figures imply that the FDA has authorized 160 drugs in the last three years (2018–2020), thereby consolidating the ascendant trend initiated in 2005 with the approval of only 20 drugs, except 2016 in which only 22 drugs were approved (Figure 1) and confirming our earlier expectations and those of other analysts regarding this positive tendency in the number of authorized drugs [2,3,4]. It is important to highlight that 2020 has been an anomalous year because it has reaffirmed the strength and solidity of both the pharmaceutical industry and regulatory agencies.

The 53 new drugs of this year comprise 40 New Chemical Entities (NCEs) (38, 42, and 34 in 2019, 2018, and 2017, respectively) and 13 biologics (10, 17, and 12 in 2019, 2018, and 2017, respectively), both figures consistent with the number of drugs approved during the last three years (Figure 1). This year, biologics continue to account for around 25% of all drugs accepted by the FDA. Since 2014, when this class of drugs reached double-digits, 82 biologics have been approved out of a total of 302 drugs, thus accounting for 27%. 

In 2020 and in parallel to the biologics approved by the CDER, the Center for Biologics Evaluation and Research (CBER) has added several new approvals [5]. Of these, the two COVID-19 vaccines have acquired special relevance. Thus, with one week’s difference (December 11 and 18), the FDA issued emergency use authorization (EUA) to the Pfizer-BioNTech COVID-19 Vaccine [6] and the Moderna COVID-19 Vaccine to tackle the global pandemic [7]. 

## 2. Discussion

Thirteen biologics were approved in 2020 as shown in Table 1, of which ten were monoclonal antibodies (mAbs), two ADCs, and one a protein (Table 1).

As in previous years since 2014, 2020 has continued to witness the prevalence of mAbs over all types of drugs—chemical entities and the remaining biologics. Indeed, mAbs account for approximately 20% (10 vs. 53) of the total number of drugs authorized this year. In addition to the mAbs listed in Table 1, in November 2020, the FDA issued two EUAs for mAbs (bamlanivimab [8] and the combination of casirivimab and imdevimab [9]) for the treatment of mild-to-moderate COVID-19 infection.

As in 2018 and 2019, the medical targets of mAbs in 2020 continue to expand out from cancer and anti-inflammatory treatments, which were the first applications of these drugs. Thus, in this year’s harvest, there are three mAbs targeting eyes diseases (two neuromyelitis optica and one thyroid eye disease), two more for the Ebola virus disease (one is a mixture of three mAbs), and one for the prevention of migraine. Considering that mAbs were also approved in recent years for the treatment of blood diseases, osteoporosis, psoriasis, nocturnal uremic syndrome, multi-drug resistant HIV, and genetic diseases, among others, these molecules are emerging as all-round drugs. 

In our previous reports, we anticipated an increase in the number of antibody-drug conjugates (ADCs) [2] approved by the FDA. Consist with our prediction, this year two more such drugs have been added to the list, reaching a total of nine ADCs to date. All these drugs are used to treat different types of cancer. 

**Figure 2:**
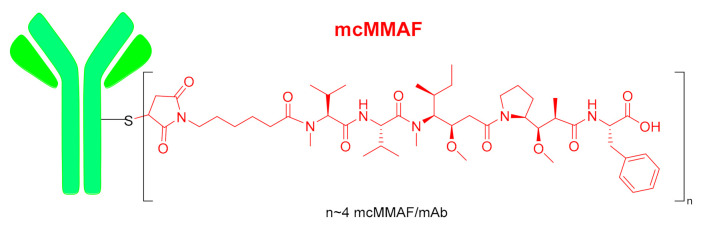
Structure of belantamab mafodotin-blm.

Belantamab mafodotin-blm (Blenrep^TM^) (Figure 2) contains the drug monomethyl auristatin F (MMAF) as a payload, which is a synthetic analog of dolastatin 10 peptide isolated from a marine mollusk. MMAF is very closely related to monomethyl auristatin E (MMAE), where the C-terminal residue, (1S,2R)-2-amino-1-phenylpropan-1-ol, in MMAE has been replaced by L-Phe. MMAE is the cytotoxic molecule contained in enfortumab vedotin-ejfv (Padcev^TM^), polatuzumab vedotin (Polivy^TM^) and brentuximab vedotin (Adcetris^TM^). Padcev^TM^ and Polivy^TM^ were approved by the FDA in 2019 and Adcetris^TM^ in 2011. In 2020, another drug, lurbinectedin (Zepzelca ^TM^), based on a marine natural product has also received approval from the FDA, thus highlighting the importance of the sea as a source of drugs. In belantamab mafodotin-blm, the conjugation between the humanized anti-B cell maturation antigen (BCMA) monoclonal antibody and MMAF is via a maleimide-based non-cleavable linker through a Michael addition of the Cys present in BCMA. In the three previous MMAE-based ADCs, the mAb is also linked through the Cys to a maleimide-based linker. This linker is bound to MMAE via a cleavable dipeptide Val-Cit linker, which contains the auto-inmolative moiety p-aminobenzyl alcohol carbamate (PABC) at the C-terminus.

**Figure 3:**
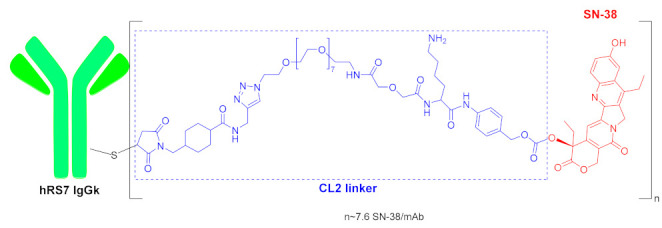
Structure of sacituzumab govitecan-hziy.

The second ADC approved this year is sacituzumab govitecan-hziy (Trodelvy^TM^) (Figure 3). The two parts of this ADC, namely the mAb and cytotoxic moiety, are bound through a Click reaction that renders a triazole. As in the previous ADC, the mAb is linked through a Michael addition of the Cys present in the mAb on a maleimide moiety. The payload drug is SN-38, the active metabolite of irinotecan, which is a topoisomerase inhibitor. This part of the conjugate contains a PABC moiety bound to the carboxylic acid of a Lys, thus forming a cleavable linker. 

The only protein approved this year is somapacitan-beco (Sogroya^TM^), a human growth hormone (hGH) analog. It has 191 residues and is produced by recombinant DNA technology in *Escherichia coli*. In contrast to other hGHs, somapacitan-beco is administered once a week and not daily. 

TIDES (oligonucleo- and pep-TIDES) could be framed among biologics and the so-called small molecules. Although TIDES have a very similar structure to biologics, they are chemically synthesized and their purity is defined by chemical parameters, and therefore they are chemical entities. Like last year, 2020 has been an excellent year for TIDES. Thus, in addition to the presence of MMAF in one ADC, three more peptides and two oligonucleotides received approval from the FDA. This means that around 10% of the new drugs authorized this year by the FDA are TIDES. 

**Figure 4:**
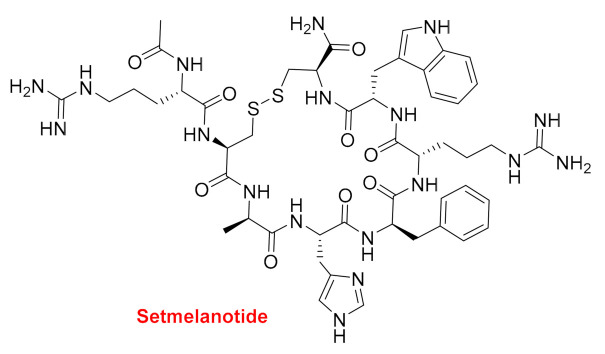
Structure of setmelanotide.

Setmelanotide (Imcivree^TM^) (Figure 4) is a disulfide cyclic octapeptide with an acetylated N-terminus and amidated C-terminus (Cys). The Ala and Phe residues are of the D configuration and the rest L configuration (Figure 4). Setmelanotide is indicated for the treatment of obesity and hunger caused by pro-opiomelanocortin lack of control. Due to several severe side effects, this drug is not recommended as a general anti-obesity agent. It just acts as a selective agonist of the hypothalamic melanocortin-4 receptor (MC4R). 

**Figure 5:**
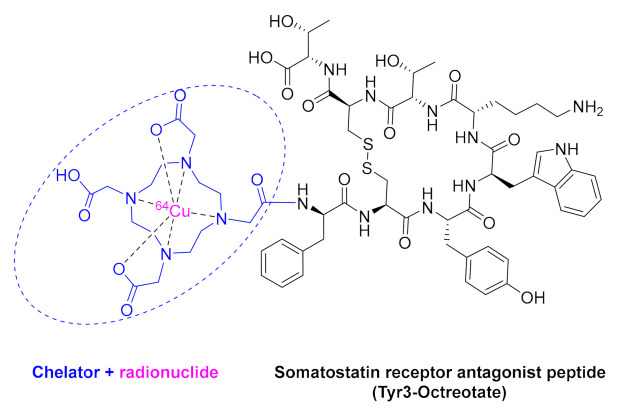
Structure of Cu-64 dotatate.

Two radioactive peptide-based diagnostic agents were approved this year. Copper Cu-64 dotatate (Detectnet^TM^) (Figure 5) contains the cyclic Tyr^3^-octreotate (TATE), which is a somatostatin receptor antagonist disulfide-based cyclic octapeptide ending at the C-terminus with Thr and at the *N*-terminus with a dodecanetetraacetic acid (DOTA) chelator, to which Cu-64 is bound. It is indicated for use with positron emission tomography (PET) for the localization of somatostatin receptor (SSR)-positive neuroendocrine tumors (NETs). This is the first FDA-approved Cu 64-labeled radiopharmaceutical for PET/computed tomography (CT) imaging. In 2019, gallium Ga-68 DOTA-TOC, which is very similar to Detectnet^TM^, with Ga-68 instead of Cu-64 and threoninol instead of Thr, was approved to measure the density and whole-body biodistribution of tumor SSRs by PET. 

**Figure 6:**
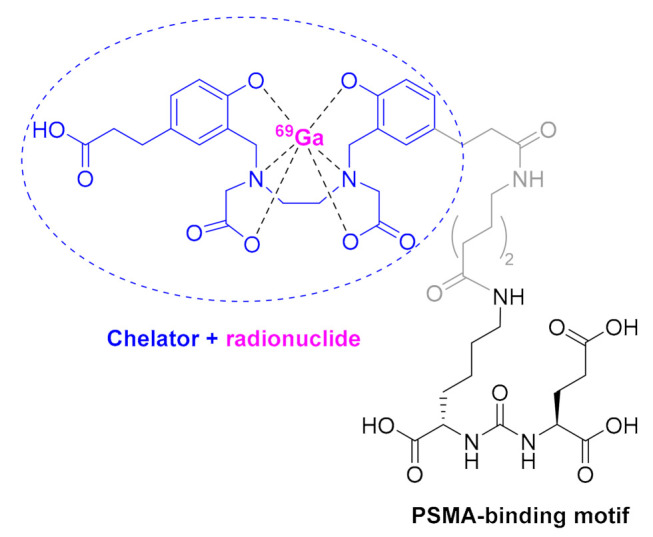
Structure of Ga-68 PSMA-1.

The second radioactive agent is gallium Ga-68 PSMA-1 (Figure 6), which is the first drug approved for PET imaging of prostate-specific membrane antigen (PSMA)-positive lesions in men with prostate cancer. PSMA is overexpressed in 90% of prostate cancers. This radiolabeling agent is formed by a PSMA-specific pharmacophore linked to the chelator *N,N*′-bis [2-hydroxy-5-(carboxyethyl)benzyl]ethylenediamine-*N,N*′- diacetic acid (HBED-CC) through an 6-aminohexanoyl (Ahx) residue. The pharmacophore is a peptidomimetic formed by a urea of the two α-amino functions of L-Glu and L-Lys, the ε-amino function of the Lys being the anchoring site to Ahx. This is the first radioactive peptide drug that is not based on a somatostatin receptor and on a DOTA chelator, thereby reflecting the expansion of this medicinal approach.

In 2016, the FDA approved three oligonucleotide-based drugs. This harvest constituted a breakthrough for this class of drugs because only three such drugs were approved between 1998 (when the first one entered the market) and 2013. This trend was confirmed by the approval of two oligonucleotides in both 2018 and 2019. Again, in 2020, two more oligos have been added to the list, making a total of 12 oligonucleotides given the green light by the FDA and confirming that the pharmaceutical industry is staking heavily on oligos as drugs. As in 2019, two oligos, one phosphorodiamidate morpholino oligomer (PMO) and one a double-stranded short-interfering ribonucleic acid (siRNA), were approved in 2020. 

**Figure 7:**
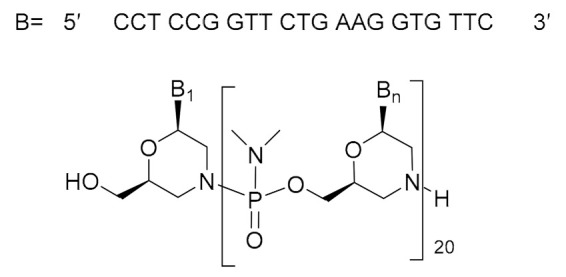
Structure of viltolarsen.

Viltolarsen (Viltepso^TM^) (Figure 7), an antisense 21 mer PMO, was authorized for the treatment of Duchenne muscular dystrophy (DMD). Viltolarsen is the third PMO approved by the FDA after eterlipsen and golodirsen in 2016 and 2019, respectively, also for the treatment of DMD. While viltolarse and golodirsen are for patients with genetic mutations subject to exon 53 skipping of the dystrophin gene, eterlipsen is for those skipping exon 51. The sequence of viltolarsen is identical to that of golodirsen except that it is missing the four residues of the 5′ end as well as the triethylene glycol moity. In contrast, the sequence of eterlipsen is unrelated to the other two PMOs. 

**Figure 8:**
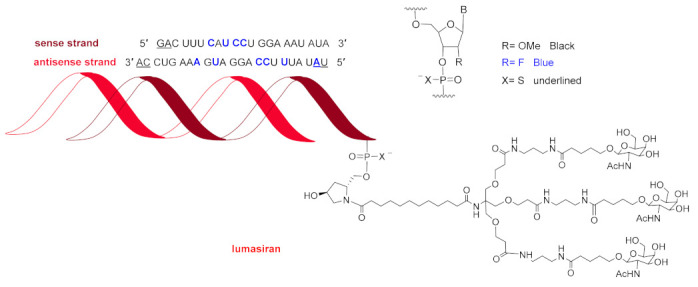
Structure of lumasiran.

Lumasiran (Oxlumo ^TM^) (Figure 8) is indicated for the treatment of hyperoxaluria type 1, a rare disorder caused by the accumulation of oxalate in the kidneys and urinary tract and resulting in severe kidney damage. Lumasiran is a double-stranded siRNA—with 21 and 23 ribonucleosides for the sense and antisense, respectively—with a chemical structure similar to that of givosiran. The latter was approved last year for acute hepatic porphyria, a genetic disorder that causes the buildup of toxic porphyrin molecules, which are formed during the production of heme. Lumasiran has a total of six tiophosphate linkages—the same as givosiran—as well as 10 2′-F-ribonucleoside units (16 in givosiran) to improve the stability of the double-strand. The remaining ribonucleosides are 2′-methoxy. Like givosiran, lumasiran is presented through an Enhanced Stabilization Chemistry (ESC), which, in addition to F- and methoxy-ribonucleosides, has the 3′ end of the sense strand linked to a short dendrimer bearing N-acetylgalactosamine (GalNAc), which mediates its binding and internalization by hepatocytes. The six thiophosphates are present in the other three ends—two in each. 

**Figure 9:**
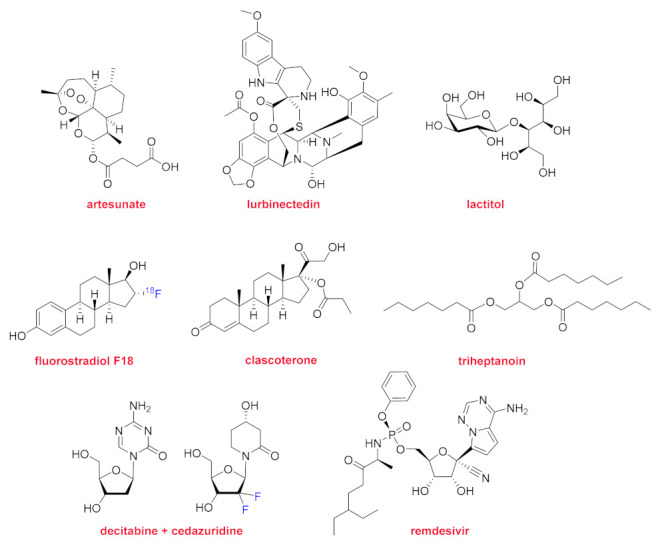
Structure of the natural product-based drugs (in blue fluorine atoms).

Regarding natural product-based drugs or those directly inspired in natural products, 2020 could provide a master class in natural product medicinal chemistry. In this regard, two steroids, and one triglyceride, sugar, nucleoside, nucleotide, and two complex molecules with several chiral centers and cycles have been approved by the FDA (Figure 9). 

Artesunate (Artesunate^TM^) and lurbinectedin (Zepzelca^TM^) are both semi-synthetic derivatives of two natural products and clear examples that without inspiration on natural products, the preparation of such complex molecules would be never have been attempted by even the most skillful chemists working in organic synthesis. Indicated for the treatment of severe malaria, artesunate is prepared by reacting dihydroartemisinin with succinic acid anhydride in a basic medium. Lurbinectedin is structurally similar to trabectedin (Yondelis^TM^) (used for the treatment of advanced soft-tissue sarcoma and ovarian cancer), which was isolated from the sea squirt *Ecteinascidia turbinate*. Lurbinectedin has been approved for metastatic small cell lung cancers. 

Lactitol (Pizensy^TM^) was already used as a sweetener and an excipient in some drugs. It has been approved for the treatment of chronic idiopathic constipation. Lactitol (β-galactoside sorbitol) is produced by lactose hydrogenation. 

In the steroid family, two drugs have been approved, fluoroestradiol F18 (Cerianna^TM^) and clascoterone (Winlevi^TM^). The former, [18F]16α-fluoroestradiol, is a radiopharmaceutical for PET imaging to detect estrogen receptor-positive breast cancer lesions. 

Triheptanoin (Dojolvi^TM^), which could be considered as a triglyceride from a chemical perspective, is indicated for the treatment of inherited metabolic diseases associated with long-chain fatty acid oxidation. 

Inqovi^TM^ is the only combination drug to be approved in 2020. Previous years witnessed the authorization of more drugs containing more than one API. Inqovi^TM^ contains two nucleoside-inspired APIs, namely decitabine and cedazuridine, and it is indicated for the treatment of patients with myelodysplastic syndromes. 

The approval of remdesivir (Veklury^TM^) could be considered unique in this unprecedented year. Remdesivir is a broad-spectrum antiviral that was initially developed for the treatment of hepatitis C and respiratory syncytial virus (RSV), but it failed to demonstrate efficacy. It was then repurposed to fight against Ebola virus disease and Marburg virus infection and showed promising preliminary results. As a result of the COVID-19 pandemic, and after observations that the median time to recovery of patients was substantially less for those taking remdesivir compared with the placebo group, this drug received EUA from the FDA in May 2020 and received official authorization as a drug in October 2020. However, there is still considerable controversy regarding its true efficacy and therefore its use. 

**Figure 10:**
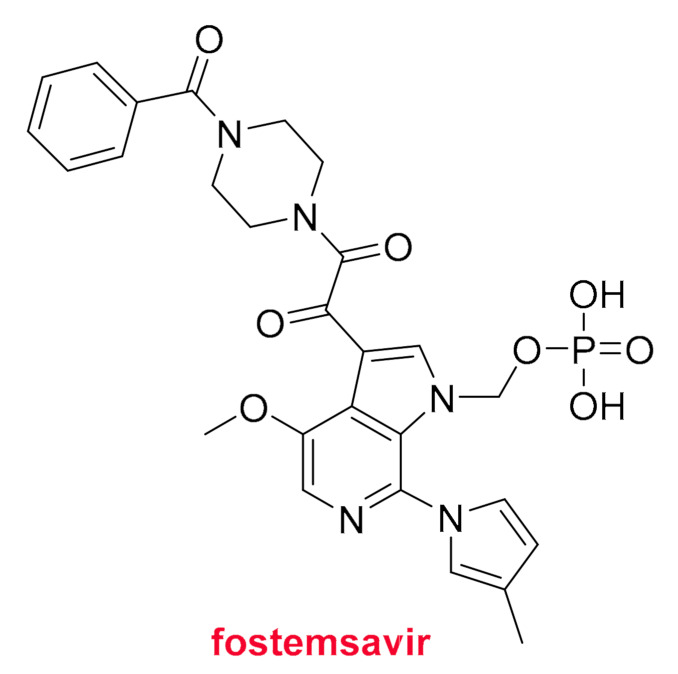
Structure of fostemsavir.

Fostemsavir (Rukobia ^TM^) (Figure 10) is the second phosphorus (V) small molecule to be approved this year. It is indicated for the treatment of patients with HIV who have shown intolerance or resistance to other therapies. Fostemsavir is a prodrug of temsavir, which has been through clinical phases. 

As in recent years, many drugs authorized in 2020 contain nitrogen aromatic heterocycles and/or fluorine atoms. These two chemical distinctives are extremely common in the drug harvest. Thus, in addition to lumasiran, fluoroestradiol F18, and cedazuridine, another 11 drugs contain fluorine. Therefore, approximately 25% (14 out of 53) of all drugs approved in 2020 by the FDA contain this atom. This percentage increases by more than a third (14 out of 40) if only NCEs are taken into consideration. These figures highlight the clear impact of fluorine in drug discovery. 

**Figure 11:**
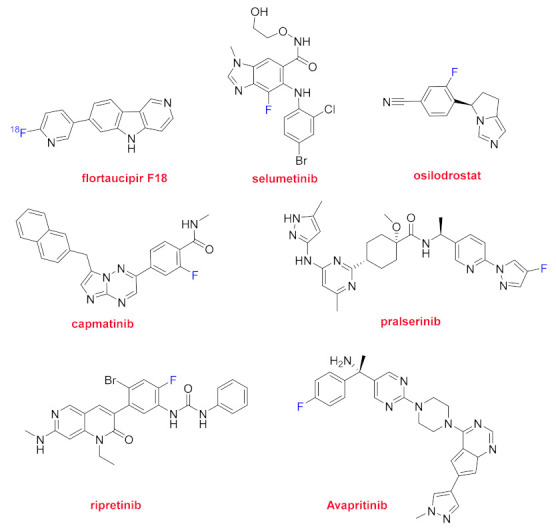
Structure of drugs containing one fluoroaryl moiety (fluorine atoms shown in blue).

**Figure 12:**
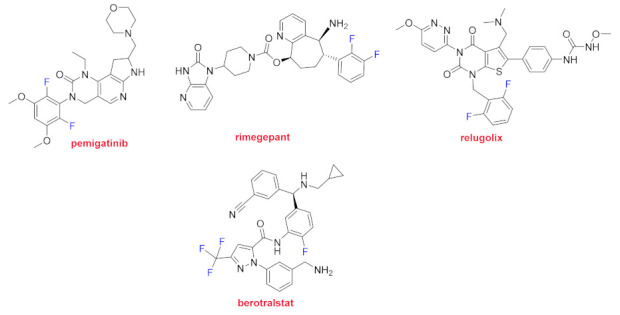
Structure of drugs containing more than one fluorine (fluorine atoms shown in blue).

The additional 11 drugs all contain fluorophenyl moieties and only one also a trifluoromethyl group (Figure 11 and Figure 12). 7-(6-(Fluoro)pyridin-3-yl)-5H-pyrido[4,3-b]indole binds to sites associated with tau protein misfolding. Flortaucipir F18 (Tauvid^TM^), the radiolabeled version of that compound, is used for brain imaging and is indicated for patients with Alzheimer’s disease. Flortaucipir F18 is the first approved diagnostic agent for imaging tau neurofibrillary tangles in the brain. Selumetinib (Koselugo^TM^) is the first drug approved by the FDA for the treatment of neurofibromatosis type 1, a genetic disorder that causes tumor growth on nerves. It is a selective inhibitor of mitogen-activated protein kinase (MAPK). Osilodrostat (Isturisa^TM^) is indicated for the treatment of adults with endogenous Cushing’s disease. It acts as a potent inhibitor of aldosterone synthase (CYP11B2). Capmatinib (Tabrecta^TM^) is indicated for the tumors of metastatic non-small cell lung cancer (NSCLC) that have a mutation that leads to mesenchymal-epithelial transition (MET) exon 14 skipping. This is the first FDA-approved therapy for NSCLC involving mutation. Capmatinib is a tyrosine kinase. In addition to fluorine, these last three drugs also contain an imidazole moiety. Pralsetinib (Gavreto^TM^) is also a tyrosine kinase inhibitor and is also approved for NSCLC, in this case for metastatic rearranged during transfection (RET) fusion-positive patients. Furthermore, pralsetinib has also been approved for metastatic RET-mutant medullary thyroid cancer. Finally, in this section of mono flurophenyl-containing drugs (Figure 10), there are two kinase inhibitors for the treatment of gastrointestinal-stromal tumors (GIST), namely, ripretinib (Qinlock^TM^) and avapritinib (Ayvakit^TM^).

Pemigatinib (Pemazyre^TM^) and rimegepant (Nurtec ODT^TM^) both contain two fluorines linked to the phenyl ring. A kinase inhibitor, pemigatinib, is indicated for metastatic or advanced cholangiocarcinoma, which is a bile duct cancer. Rimegepant is a calcitonin gene-related peptide receptor antagonist for the treatment of severe migraine. Relugolix (Orgovyx^TM^) is a gonadotropin-releasing hormone (GnRH) antagonist used for the treatment of advanced prostate cancer. In addition to a fluorophenyl moiety, Berotralstat (Orladeyo^TM^) contains a trifluoromethyl group and it is used to prevent attacks of hereditary angioedema (HAE) (Figure 12). 

**Figure 13:**
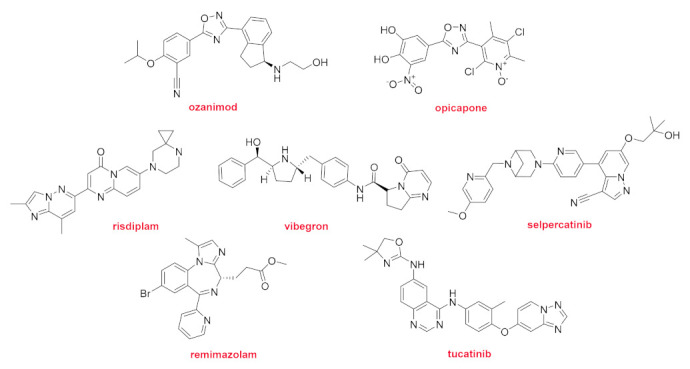
Structure of drugs containing nitrogen aromatic heterocycles.

**Figure 14:**
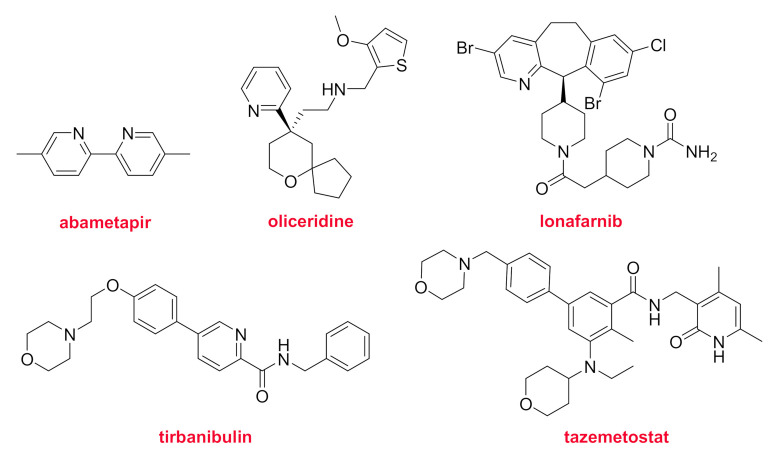
Structure of drugs containing pyridine moieties.

In addition to the 16 drugs that contain nitrogen aromatic heterocycles previously discussed, FDA authorization has been given to another 12 drugs with the same characteristics (Figure 13 and Figure 14). This means that approximately 50% (28 out of 53) of the drugs approved this year belong to that class. If biologics and peptides are not considered, three out of every four drugs approved in 2020 (28 out of 37) contain nitrogen aromatic heterocycles. Two of these structures without fluorine atoms contain an oxadiazole ring. The immunomodulatory drug Ozanimod (Zeposia^TM^) is the only FDA-approved sphingosine-1-phosphate (S1P) receptor modulator indicated for the treatment of relapsing forms of multiple sclerosis (RMS). When administered together with levodopa (L-DOPA), opicapone (Ongentys^TM^) restores the levels of dopamine in the regions of the brain that control movement and coordination. Two more drugs contain a pyrimidinone moiety. Risdiplam (Evrysdi^TM^) is the first oral drug approved for the treatment of spinal muscular atrophy (SMA). Risdiplam also contains a pyridazine that modifies the splicing of a messenger RNA, thereby increasing the concentration of the functional survival motor neuron (SMN) protein in vivo. Vibegron (Gemtesa^TM^), which is a β_3_-adrenergic receptor (AR) agonist, is recommended for the treatment of overactive bladder. Remimazolam (Byfavo^TM^) is a benzodiazepine derivative used for the induction and maintenance of sedation. Tucatinib (Tukysa^TM^) is an inhibitor of HER2 used for the treatment of HER2-positive breast cancer. Selpercatinib (Retevmo^TM^) is the third drug approved this year for NSCLC and other RED fusion-positive thyroid cancers.

Five drugs containing pyridine as nitrogen aromatic heterocycle have received FDA approval in 2020 (Figure 14). From a structural point of view, the simplest drug authorized this year is probably abametapir (Xeglyze^TM^), a dimethyl-bipyridine. Abametapir is a metalloproteinase inhibitor for the treatment of head lice infestation. Oliceridine (Olinvyk^TM^) is a G protein-selective agonist at the μ-opioid receptor. It is structurally unrelated to morphine and is recommended for the treatment of moderate to severe acute pain. Lonafarnib (Zokinvy^TM^) is for Hutchinson-Gilford progeria syndrome and some progeroid laminopathies, which are genetic diseases that result in premature aging and even death. Tirbanibulin (Klisyri^TM^) shows a relatively simple structure and it is indicated for the treatment of actinic keratosis of the face or scalp. It is a first-in-class microtubule inhibitor. Tazemetostat (Tazverik ^TM^) is recommended for metastatic and advanced epithelioid sarcoma. It is a selective inhibitor of Enhancer of zeste homolog 2 (EZH2).

**Figure 15:**
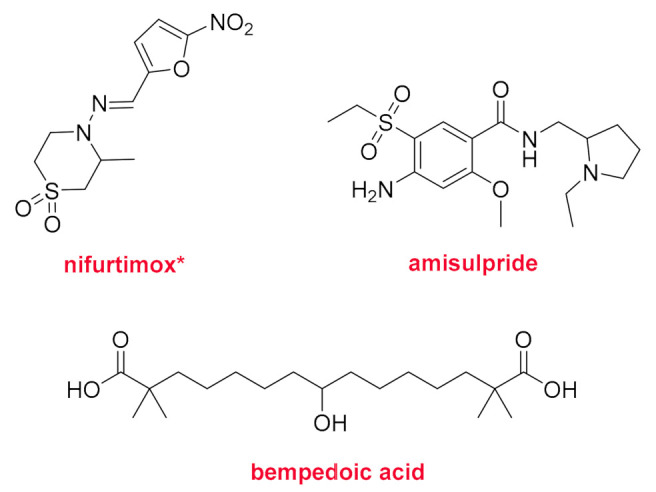
Structure of nifurtimox, amisulpride, and bempedoic acid. * Mixture of Z/E conformers

Nifurtimox (Lampit^TM^) (Figure 15) is a nitrofuran drug for the treatment of Chagas disease in young patients. Although used since 1965, it has not been formally approved by the FDA until this year. The nitro-anion radical metabolite from nifurtimox breaks down the parasite DNA. Amisulpride (Barhemsys^TM^), which is a benzamide, was developed in the 20th century and has multiple indications (schizophrenia, acute psychotic episodes, dysthymia). This year, the FDA has authorized the use of this drug for the prevention of nausea and vomiting after surgery. The last drug to receive the green light in this prolific 2020 is bempedoic acid (Nexletol^TM^), which is indicated for the treatment of familial hypercholesterolemia and for those patients requiring additional lowering of LDL-C due to atherosclerotic cardiovascular disease. It is an odd fatty diacid that forms a thioester with coenzyme A, which inhibits the ATP citrate lyase involved in the biosynthesis of cholesterol.

## 3. Conclusions

There is widespread consensus among society at large and the pharmaceutical industry ecosystem that 2020 will go down as the year of COVID-19. Only about one year has elapsed between the detection of the disease and the rollout of the first vaccinations. Although the public may consider this a long time, those working in the health arena know that it is a ridiculous fraction of the time usually required for the development and authorization of a vaccine. Indeed, this short period saw the detection of the disease, the approval of two first-in-class vaccines, with complex production and transportation logistics, and the mass rollout of these vaccines in North America, Europe, and other parts of the world. It is expected that a third vaccine will be approved in the first quarter of 2021. These achievements reflect the strength and the solidity of the pharmaceutical ecosystem, which includes biotech companies, academic groups, big pharma companies, contract research and manufacturing organizations, and, last but not least, the regulatory agencies. An important question arises in the context of the achievements made in 2020 in the context of COVID-19, namely will the events of this year have a positive impact on regulatory agency approval of new drugs, diagnostics, vaccines, and other biologics? If this occurs, time and money will be saved, thereby auguring a better future for society.

As reflected throughout this report, 2020 has been an excellent year for the FDA approval of drugs, confirming the trend of the last three years, which averaged the authorization of more than 50 drugs each year. Together, the last three years make history in terms of the number of drugs to be given the green light by the FDA. Again, analysts are very cautious when talking about drugs launched into the market, but the general trend augurs a propitious future for the sector [2,3,4].

**Figure 16:**
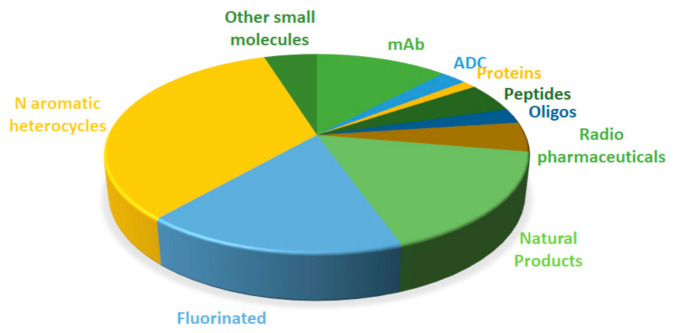
Drugs approved by the FDA in 2020 classified on the basis of their chemical structure (drugs can belong to two distinct classes).

Figure 16 shows the drugs approved by the FDA in 2020 classified on the basis of their chemical structure.

The harvest of TIDES and mAbs in 2020 has been an almost carbon copy of 2019 [2]. In 2019, a double-stranded siRNA drug, givosiran, and in 2020 other one, lumasiran, were approved. Both share the same delivery approach through the so-called “enhanced stabilization chemistry (ESC)”. These two drugs, together with patisiran from 2018, brings a total of three of these intriguing drugs into the market. Importantly, these three drugs are indicated for distinct medical targets. All of these data confirm our statement two years ago that the approval of the first one “opens the door for the authorization of others”. In 2019, the antisense PMO golodirsen was approved. In 2020, it was the turn of viltolarsen. These two drugs, together with eterlipsen (approved in 2016), bring a total of three of this second class of oligonucleotides into the market. In this regard, we can repeat the same statement highlighted for double-stranded siRNA drugs, in that more will come in the next years.

Three peptides were approved in both 2019 and 2020. Three of these six belong to the class of radiolabeled pharmaceuticals, thereby revealing the relevance of peptides for directing the radioagent moiety. 2020 has witnessed the approval of two more of these compounds but based on small molecules as targeting moieties. It is important to highlight the case of flortaucipir F18, which is used for imaging patients with Alzheimer’s disease. As the first approved diagnostic agent to image tau neurofibrillary tangles in the brain, we consider that flortaucipir F18 deserves the title of “Molecule of the Year 2020”.

While three ADCs were approved in 2019, this year has seen the authorization of two ADCs. Three of these five contain synthetic analogs of the sea peptide dolastatin 10 as payload.

In 2020, ten mAbs were approved, matching the numbers of nine and eleven approved in 2017 and 2018, respectively. In 2019, only five were authorized.

There is no doubt that natural products are the first source of inspiration in drug discovery, with eight approvals this year. In this regard, it is important to highlight the complexity but also the beauty of the structures of two of the drugs authorized this year, namely artesunate and lurbinectedin. Both contain rare cyclic structures (peroxide and ether in the former and ester and thioether in the latter), thereby confirming that the nature is also unbeatable as a drug designer.

The presence of fluorine- and nitrogen aromatic heterocycle-based drugs continue to be a constant. Thus, excluding biologics and peptides from the analysis, 38% of the drugs approved this year contain the first moiety and 75% the second.

As in previous years, oncology continues to be the main target of the drugs approved by the FDA in 2020—with three of them indicated for metastatic NSCLC. The broad research into radiopharmaceuticals for diagnostics carried out in recent years is paying off, with four drugs accepted this year. Remarkably, copper Cu-64 dotatate is the first FDA-approved Cu 64-based radioagent. Due to the special characteristics of these pharmaceuticals, some have been developed by public-private sector partnerships.

In this forum, we commonly draw attention to the cost of treatments. Although such costs are very high (six-digit figures) in many cases, this year we would also like to comment on the prices of the two COVID-19 vaccines (each around US $20). Given that both are first-in-class drugs composed by RNA, their cost, although still unaffordable for most of the world’s population, could be considered reasonable.

## Figures and Tables

**Table 1:** Biologics approved by the FDA in 2020 [1].

Trade name ^a^	Active Ingredient ^a^	Class	Indication
Blenrep^TM^	Belantamab mafodotin-blm	Antibody-drug conjugate	Multiple myeloma
Danyelza^TM^	Naxitamab-gqgk	Monoclonal antibody	High-risk refractory or relapsed neuroblastoma
Ebanga^TM^	Ansuvimab-zykl	Monoclonal antibody	Ebola virus
Enspryng^TM^	Satralizumab-mwge	Monoclonal antibody	Neuromyelitis optica spectrum disorder
Inmazeb^TM^	Atoltivimab, Aaftivimab, Odesivimab-ebgn	Monoclonal antibodies	Ebola virus
Margenza^TM^	Margetuximab	Monoclonal antibody	HER2+ breast cancer
Monjuvi^TM^	Tafasitamab-cxix	Monoclonal antibody	Relapsed or refractory diffuse large B-cell lymphoma
Sarclisa^TM^	Isatuximab	Monoclonal antibody	Multiple myeloma
Sogroya^TM^	Somapacitan-beco	Protein	Growth hormone
Tepezza^TM^	Teprotumumab-trbw	Monoclonal antibody	Thyroid eye disease
Trodelvy^TM^	Sacituzumab govitecan-hziy	Antibody-drug conjugate	Metastatic triple-negative breast cancer
Uplizna^TM^	Inebilizumab-cdon	Monoclonal antibody	Neuromyelitis optica spectrum disorder
Vyepti^TM^	Eptinezumab-jjmr	Monoclonal antibody	Preventive treatment of migraine in adults

^a^ Trade name used in the USA.

## References

[1] U.S. Food and Drug Administration (FDA) https://www.fda.gov/drugs/new-drugs-fda-cders-new-molecular-entities-and-new-therapeutic-biological-products/novel-drug-approvals-2020.

[2] De La Torre B.G., Albericio F. (2020). The Pharmaceutical Industry in 2019. An Analysis of FDA Drug Approvals from the Perspective of Molecules. Molecules.

[3] Mullard A. (2020). 2019 FDA drug approvals. Nat. Rev. Drug Discov..

[4] Jarvis L.M. (2020). The new drugs of 2019. Chem. Eng. News.

[5] U.S. Food and Drug Administration (FDA) https://www.fda.gov/vaccines-blood-biologics/development-approval-process-cber/2020-biological-license-application-approvals.

[6] U.S. Food and Drug Administration (FDA) https://www.fda.gov/emergency-preparedness-and-response/coronavirus-disease-2019-covid-19/pfizer-biontech-covid-19-vaccine.

[7] U.S. Food and Drug Administration https://www.fda.gov/emergency-preparedness-and-response/coronavirus-disease-2019-covid-19/moderna-covid-19-vaccine.

[8] U.S. Food and Drug Administration (FDA) https://www.fda.gov/news-events/press-announcements/coronavirus-covid-19-update-fda-authorizes-monoclonal-antibody-treatment-covid-19#:~:text=Monoclonal%20antibodies%20are%20laboratory%2Dmade,and%20entry%20into%20human%20cells.

[9] U.S. Food and Drug Administration (FDA) https://www.fda.gov/news-events/press-announcements/coronavirus-covid-19-update-fda-authorizes-monoclonal-antibodies-treatment-covid-19.

